# Modified commando procedure in complicated infective endocarditis ─ a case series

**DOI:** 10.1186/s13019-021-01480-4

**Published:** 2021-04-13

**Authors:** Posung Chen, Chungyi Chang, Yicheng Chuang, Ichen Chen, Tingchao Lin

**Affiliations:** 1 Division of Cardiovascular Surgery, Heart Center, Cheng Hsin General Hospital, Taipei, Taiwan; 2 Department of Surgery, Kinmen Hospital, Kinmen, Taiwan; 3 Department of Medicine, School of Medicine, National Yang-Ming University, Taipei, Taiwan

**Keywords:** Infective endocarditis, Intervalvular fibrous body, Prosthetic valve endocarditis, Valvular heart disease, Surgical reconstruction

## Abstract

**Background:**

Complicated infective endocarditis (IE) with perivalvular abscess and destruction of intervalvular fibrous body (IFB) has high mortality risk and requires emergent or urgent surgery mostly.

**Case presentation:**

We presented four patients with complicated infective endocarditis combined with perivalvular abscess and IFB destruction. Three patients had prosthetic valve endocarditis and one patient had native valve endocarditis. They all received modified Commando procedure successfully. No surgical mortality or re-exploration for bleeding.

**Conclusions:**

We suggest that modified Commando procedure may have some benefit in improving survival rate of patients with complicated IE and reducing complications.

## Introduction

Complicated infective endocarditis (IE) with perivalvular abscess and destruction of the intervalvular fibrous body (IFB) has a high mortality risk and mostly requires emergent or urgent surgery. David et al. [1] reported the technique of aortic valve replacement (AVR), mitral valve replacement (MVR) combined with reconstruction of the IFB, which is known as the Commando procedure. However, a high mortality rate and re-exploration for bleeding were noted in early series, which might be related to the emergent surgical procedure, fragile infected tissue, and difficulty of achieving hemostasis due to inaccessible posterior suture lines. The complicated condition is sometimes inevitable, but we would like to introduce a modified Commando procedure, which may improve surgical exposure, solidify the infected tissue, decrease tension, and transfer the suture line to the anterior aspect for better hemostasis. Here, we describe the cases of 4 patients who underwent reoperation due to prosthetic IE with IFB involvement and the surgical technical modifications we introduced to the origin procedure.

### Surgical technique of modified commando procedure

**Fig. 1:**
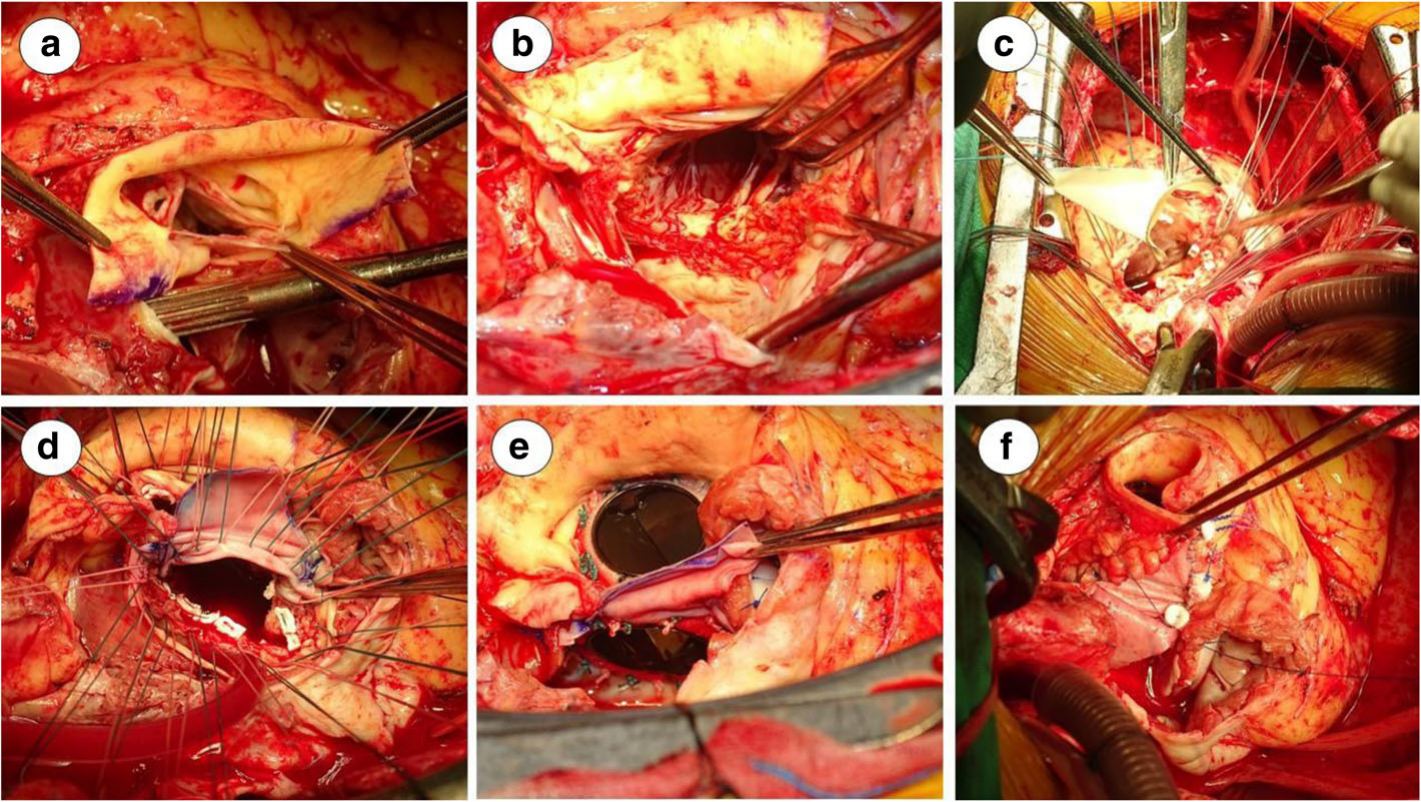
**a** Extend the aortotomy from the noncoronary cusp into the IFB and onto the left atrial roof. **b** Post removal of infective tissue (aortic valve, mitral valve, partial aortic annulus and IFB). **c** Bovine pericardium (two triangular shape) for reconstruction of IFB, interatrial septum and aortic root. **d** Post IFB reconstruction. Suture for valve replacement. **e** Post AVR and MVR. **f** Reconstruction of interatrial septum and aortic root, then proceed to reconstruction of left atrial roof and right atrium

1. Cardiopulmonary bypass was established via the ascending aorta and bicaval cannulation. Custodiol HTK cardioplegic solution was used.
2. Surgical exposure was achieved through oblique aortotomy and an extended transseptal approach. The aortotomy was extended from the noncoronary cusp into the IFB and onto the left atrial roof (Fig. 1a).
3. Inspect the aortic valve area and the mitral valve area. Remove all infected tissues (Fig. 1b), and the remaining margin was treated with glutaraldehyde solution carefully.
4. Reconstruction of the IFB was performed with one diamond-shaped patch of bovine pericardium folded into two triangular shapes (Fig. 1c), one for reconstruction of the interatrial septum and the other for reconstruction of the aortic root.
5. Perform MVR and AVR, suture anchoring at the remaining margin and the new IFB (Fig. 1d & e).
6. Reconstruction of the aortic root and the interatrial septum (Fig. 1f).
7. Another bovine pericardium patch was used for reconstruction of the left atrial roof and right atrium to close the opening of the extended transseptal approach.
8. Weaning cardiopulmonary bypass.

#### Case

##### Patient 1

A 46-year-old woman was admitted to our hospital due to intermittent dyspnea on exertion for 2 months and fever for 1 week. She had undergone mitral valve repair and tricuspid valve repair (TVr) 8 years prior due to IE with symptomatic severe mitral regurgitation (MR) and tricuspid regurgitation. Transthoracic echocardiography (TTE) revealed moderate to severe aortic regurgitation (AR), severe MR and vegetation over the anterior mitral leaflet that extended to the left ventricular outflow tract (LVOT). Recurrent IE was diagnosed. Antibiotic therapy was initiated, but intermittent fever persisted, so urgent surgery was arranged.

**Fig. 2:**
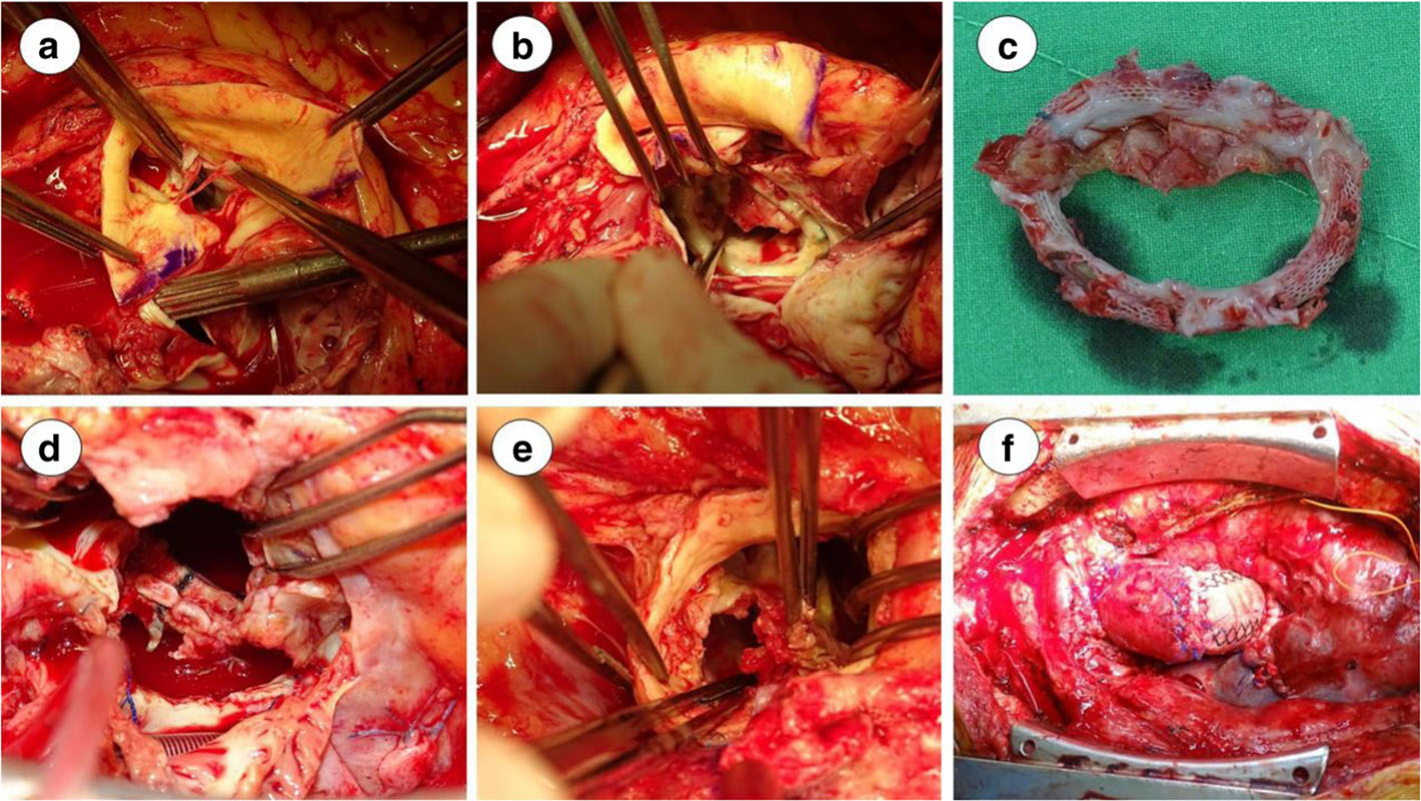
**a** Patient 1, aortic valve perforation: non-coronary-cusp & left-coronary-cusp. **b** Patient 1, mitral valve post annuloplasty with vegetable formation and perforation. **c** Patient 1, post removal of mitral valve and annuloplasty ring. **d** Patient 2, dehiscence of mitral prosthetic valve. **e** Patient 3, vegetations over aortic perivalvular area and IFB perforation. **f** Patient 3, post modified Commando procedure and aortic root reconstruction with Cabrol method

In the aortic valve area, left-coronary-cusp and noncoronary-cusp perforation with perivalvular abscess formation over the aortic root was observed (Fig. 2a). In the mitral valve area, vegetation was observed on the anterior mitral leaflet extending to the IFB and LVOT (Fig. 2b). Mitral valve and annuloplasty ring were removed (Fig. 2c). The modified Commando procedure was performed during the reoperation.

The postoperative course was uneventful except that a permanent pacemaker (PPM) was implanted for complete heart block. There were no other complications and no restrictions on physical activity after discharge. Follow-up TTE examinations at 1 year and 3 years revealed good valve function, and no paravalvular leakage was noted.

##### Patient 2

A 41-year-old man was admitted to our hospital for decompensated heart failure. He had undergone MVR and TVr 3 months prior and anal fistulectomy 1 month prior. TTE revealed dislocation of the mitral prosthetic valve and severe right ventricular dysfunction with an ejection fraction of 28%. At surgery, vegetation on the aortic valve, mitral prosthetic valve and IFB was detected. Nearly total dehiscence of the mitral prosthetic valve and posterior atrial-ventricular groove containing rupture was also detected (Fig. 2d). The ruptured atrial-ventricular groove was repaired with one bovine pericardium patch, and the modified Commando procedure was performed. A PPM was implanted postoperatively, and the residual course was uneventful.

##### Patient 3

A 60-year-old man was admitted to our hospital for heart failure and intracardiac shunt (LVOT to left atrium). He had IE and left medial frontal cerebral infarction and undergone AVR at another hospital 2 weeks prior. In addition, he had been intubated preoperatively due to respiratory failure caused by influenza A infection. During surgery, dehiscence of the aortic prosthetic valve with an annular abscess and vegetation over the aortic root to the mitral valve and one large perforation of the IFB were detected (Fig. 2e). The modified Commando procedure was performed, along with aortic root reconstruction with a metallic valve and gelatin vascular graft and the Cabrol method for coronary ostia reimplantation (Fig. 2f). The postoperative course was prolonged due to preoperative respiratory failure and poor brain infarction-related mobility. A PPM was implanted for heart block. Multiple debridement and muscular flap reconstruction procedures were performed for sternal infection. He was discharged in a wheelchair and has undergone continuous rehabilitation.

##### Patient 4

An 81-year-old woman was admitted to our hospital for fever and dyspnea for 3 days. TTE revealed aortic valve vegetation with severe AR and severe MR. IE complicated with valvular insufficiency was diagnosed. At surgery, vegetation on the aortic noncoronary cusp, LVOT and IFB, an aortic annular abscess with perforation and mitral valve anterior leaflet perforation were detected. The modified Commando procedure was performed with bioprosthetic valves due to the advanced age of the patient. The postoperative course was smooth, except PPM implantation was performed for heart block.

## Discussion

Surgical treatment for infective endocarditis involving the IFB is challenging. Among the limited studies of IFB repair, the incidence of mortality and re-exploration for bleeding is worth noting. David et al. [1] reported that 43 patients (14 with endocarditis) underwent double valve replacement and IFB reconstruction. Of the 14 patients with endocarditis, eleven had prosthetic valve endocarditis. There were three operative deaths (21.4%). Davierwala et al. [2] reported that 25 patients underwent the Commando procedure. Twenty of them had prosthetic valve endocarditis (80%). Eight patients (32%) died within 30 days of surgery, and the major cause of mortality (3 patients) was uncontrolled bleeding due to poor patch anchoring in infection-ridden and devitalized tissue. Additionally, Forteza et al. [3] reported that 26 patients with IE underwent the Commando procedure, resulting in an in-hospital mortality rate of 15.4% and a rate of re-exploration for bleeding of 15.4%.

In our dataset, 16 patients having complicated IE in total 126 patients received surgery because of IE from January 2014 to January 2021 (Table 1). The management of perivalvular lesions varied according to the extensity of IFB involvement. For localized perivalvular lesions, we prefer direct repair or simple patch repair. For those with advanced IFB and aortic annulus involvement, aortic annulus reconstruction with preservation of mitral valve is essential. Total aortic root reconstruction is only for extensive annulus destruction, and a bovine patch covering is preferred for partial annulus involvement. For the most extended situation with IFB involvement, complete IFB and aortic root reconstruction are the standard procedure in our institution. According to our result, the PPM implantation rate is significantly higher in those receiving total aortic root reconstruction and IFB reconstruction. Although the disease complexity of this case series reported is difficult, there is no mortality case in our cohort. With the modified Commando procedure, we can achieve a result similar to other less advanced IE.

**Table 1:** Complicated IE cases (2014.01–2021.01)

No.	Age/Sex	Site of IE involvement	Perivalvular manage	Operation methods	Survival	Post-operative events
1	46F	AV, MV, IFB, LVOT	Complete IFB reconstruction	Modified Commando	Y	PPM
2	41 M	AV, MV, IFB	Complete IFB reconstruction	Modified Commando	Y	PPM
3	60 M	AV, MV, IFB	Complete IFB reconstruction	Modified Commando	Y	PPM, Sternal infection
4	81F	AV, MV, IFB, LVOT	Complete IFB reconstruction	Modified Commando	Y	PPM
5	59 M	LVOT-RA fistula IFB	Complete IFB reconstruction	AVR + CABG^a^, Bovine patch: IFB + LVOT	Y	PPM, Reoperation (IE)
6	72 M	AV, aortic root-RA fistula	Aortic root reconstruction	Bentall, Bovine patch: LVOT + aortic annulus	Y	PPM, Bleeding
7	63 M	AV, MV, aortic annulus	Aortic root reconstruction	Bentall+MVR, Bovine patch: LVOT + aortic annulus	Y	PPM
8	56 M	AV, aortic annulus	Aortic root reconstruction	Bentall+MVA + TVA^b^, Bovine patch: LVOT + aortic annulus	Y	PPM
9	48 M	AV, aortic annulus	Aortic root reconstruction	Bentall+MVA + TVA^b^, Bovine patch: aortic annulus	Y	n/a
10	66 M	AV, aortic annulus	Aortic root reconstruction	Bentall, Bovine patch: aortic annulus	Y	PPM
11	29 M	Aortic root	Aortic root reconstruction	Bentall, Bovine patch: aortic annulus	Y	ECMO
12	41 M	AV, aortic annulus	Patch reinforcement	AVR, Bovine patch: aortic annulus	Y	2nd procedure^c^ (recurrent PVL)
13	66F	MV, TV, mitral annulus	Patch reinforcement	MVR + TVA, Bovine patch: mitral annulus	Y	ECMO
14	79F	AV, sinus valsalva-LA fistula	Patch reinforcement	AVR + MVR + TVA^b^, Bovine patch: sinus valsalva	Y	PPM, RF, RespF
15	63F	AV, partial IFB	Patch reinforcement	AVR, Bovine patch: partial IFB	Y	n/a
16	58 M	AV, TV, sinus valsalva-RA fistula	Patch reinforcement	AVR + TVR, Bovine patch: sinus Valsalva + aortic root	Y	PPM

*IE* infective endocarditis, *AV* aortic valve, *MV* mitral valve, *IFB* intervalvular fibrous body, *LVOT* left ventricular outflow tract, *PPM* permanent pacemaker, *RA* right atrium, *AVR* aortic valve replacement, *CABG* coronary artery bypass graft, *MVR* mitral valve replacement, *TVA* tricuspid valve annuloplasty, *ECMO* extracorporeal membrane oxygenation, *PVL* paravalvular leak, *TV* tricuspid valve, *LA* left atrium, *RF* renal failure, *ResF* respiratory failure

^a^Patient also had coronary artery disease

^b^Patient also had mitral valve disease and tricuspid valve disease

^c^Paravalvular leak closure with endovascular plug implantation

There were no cases of in-hospital mortality or re-exploration for bleeding in our series (Patient 1–4). The modification of the original Commando procedure may have contributed to these results. First, we used an extended transseptal approach, with wide opening of the right atrium, interatrial septum, left atrium, and aortic root. This approach provides better exposure than an extended aortotomy incision in the LA roof only. It also provides excellent exposure and an unobstructed approach for complete debridement and valve replacement. Second, an extra patch is used to repair the right atrium to further reduce the tension. The folded bitriangular bovine pericardium patch, which was used for left atrial roof and aortic reconstruction in the abovementioned studies, was used to reconstruct the interatrial septum and aortic root. This method may lessen the tension on the IFB and transfer the suture line from the posterior aspect of the heart to the anterior aspect, which may help to identify bleeding and facilitate hemostasis. Third, we used glutaraldehyde solution to treat the remaining tissue surface. Glutaraldehyde treatment has the benefit of eliminating bacteria and reinforcing the remaining tissue, making further sutures more stable and reducing the risk of tissue tearing and bleeding; it may also decrease the postoperative infection rate. Nakamura et al. [4] reported on 35 IE patients who underwent mitral valve surgery with glutaraldehyde treatment and concluded the safety of this glutaraldehyde treatment. However, AV nodes may be affected during the procedure, which may contribute to the high PPM implantation rate in our series. Last, the rocking motion of the mitral valve during systole and diastole is a problem [1]. We sew the noncoronary cusp part of the aortic prosthesis and the anterior part of the mitral prosthesis together to enhance the new IFB and reduce the risk of patch dehiscence or valve dehiscence. This procedure does not increase the risk of bleeding, and follow-up echocardiography does not reveal any sign of structural destruction or paravalvular leakage.

The choice of prosthesis in patients with infective endocarditis is controversial. Few studies have suggested that homografts have the benefit of preventing recurrent endocarditis [5, 6]. However, Moon et al. reported that 306 patients with IE (97 patients with prosthetic endocarditis) underwent valve replacement and that survival was independent of valve type [7]. As previous studies have suggested, [1–3] we believe that the radical debridement of infected tissue is more important than the type of valve. An aortic valve homograft with a preserved anterior leaflet of the mitral valve may be ideal in this procedure, but the uncertain availability in most urgent or emergent cases is a disadvantage. In addition, the durability is doubtful and may result in patients facing a high-risk reoperation. Therefore, we chose to use mechanical prostheses in most patients to reduce the possibility of a high-risk reoperation.

## Conclusion

Infective endocarditis involving the IFB is an urgent or emergent situation that requires immediate treatment with high mortality and morbidity rates. The modified Commando procedure as presented may improve the exposure of the surgical field, reduce the ‘rocking motion’ of the prosthetic valve, reduce tension via the extra patch, and help to achieve hemostasis by transferring the suture line from the posterior aspect to the anterior aspect. The results are optimal, with no cases of in-hospital mortality, re-exploration for bleeding, or paravalvular leakage. More extended experience and long-term surveillance are required to confirm its efficacy and long-term durability.

## Data Availability

The datasets used and analyzed during the current study are available from the corresponding author on reasonable request.

## Abbreviations

IE: Infective endocarditis
IFB: Intervalvular fibrous body
AVR: Aortic valve replacement
MVR: Mitral valve replacement
TVr: Tricuspid valve repair
MR: Mitral regurgitation
TTE: Transthoracic echocardiography
AR: Aortic regurgitation
LVOT: Left ventricular outflow tract
LA: Left atrial
PPM: Permanent pacemaker
